# A longitudinal study on the relationship between mother’s personality trait and eating behaviors, food intake, maternal weight gain during pregnancy and neonatal birth weight

**DOI:** 10.1186/s12937-020-00584-2

**Published:** 2020-07-06

**Authors:** Mahboobeh Shakeri, Sima Jafarirad, Reza Amani, Bahman Cheraghian, Mahin Najafian

**Affiliations:** 1 Nutrition and Metabolic Diseases Research Center, Ahvaz Jundishapur University of Medical Sciences, Ahvaz, Iran; 2 Department of Nutrition, School of Allied Medical Sciences, Ahvaz Jundishapur University of Medical Sciences, Ahvaz, Iran; 3 Food Security Research Center, Department of Clinical Nutrition, School of Nutrition and Food Science, Isfahan University of Medical Sciences, Isfahan, Iran; 4 Health Research Institute, Diabetes Research Center, Ahvaz Jundishapur University of Medical Sciences, Ahvaz, Iran; 5 Department of Epidemiology and Biostatistics, School of Health, Ahvaz Jundishapur University of Medical Sciences, Ahvaz, Iran; 6 Department of Obstetrics & Gynecology, School of Medicine, Ahvaz Jundishapur University of Medical Sciences, Ahvaz, Iran

**Keywords:** Pregnancy, Personality traits, Eating behavior, Food intake, Gestational weight gain, Birth weight

## Abstract

**Background:**

Many factors such as social and behavioral are related to appropriate weight gain during pregnancy, and there is much of importance to find them. The aim of the study was to explore the association of personality traits, with eating behaviors, food intake, maternal weight gain during pregnancy as well as the neonatal birth weight.

**Methods:**

This is a longitudinal and cross-sectional study. Eating behaviors were assessed using the Dutch Eating Behavior Questionnaire (DEBQ), and the NEO personality inventory was used to assess personality in pregnant subjects. A validated food frequency questionnaire was used to determine food intake. Three hundred and sixty pregnant subjects from Ahvaz (the capital city of Khuzestan province, Iran) were followed from the 20th week of pregnancy until delivery.

**Results:**

High neuroticism was associated with higher consumption of highly energetic foods (*p* < 0.05) and less consumption of vegetables (*p* < 0.01), also was related with lower weight gain during pregnancy and neonatal birth weight (*p* < 0.05). Openness to experience, extraversion and agreeableness were linked with higher consumption of vegetables (p < 0.05). Conscientiousness predicted lower neonatal weight (odds ratio: 1.20, confidence interval: 1.07–1.34, *p* < 0.01).

**Conclusions:**

Identification of personality traits would help to change the lifestyle and improve management guidelines.

## Background

According to previous studies, there is a relationship between personality traits and various health issues, such as cardiovascular diseases, high blood pressure, diabetes, gastroenterological complaints [1, 2], inflammatory responses [3, 4] and general health condition [5]. It is often assumed that the relationships between personality traits and well-being are due to some health-related behaviors such as dietary habits and various other unhealthy behaviors [2, 6, 7], for example, smoking [8], alcohol consumption [9], drug abuse [10], lack of physical exercise [11], and a number of other behaviors that influence the health [12].

Personality traits can influence lifestyle, cognition, motivation, and behavior in various conditions [13]. Understanding personality traits is important for maintaining health. The Five-Factor Model (FFM) is often used for the assessment of personality traits [14]. This model has been validated in many studies in different cultures by using different assessment tools [15–17]. FFM distinguishes five dimensions of personality: Neuroticism, Extraversion, Openness, Agreeableness, and Conscientiousness [18]. A study showed subjects with higher body mass index (BMI) had lower scores on conscientiousness or higher scores on extraversion or neuroticism personality traits. A similar relationship was also shown for body fat, as well as waist, and hip circumference [7]. Other studies consider neuroticism as a risk factor for obesity and showed protective effect of conscientiousness toward weight gain [19].

Various mechanisms contribute to the relationship between personality and body weight, which include cognitive, behavioral, physiological and psychological pathways [12]. In recent studies, personality traits have been found to be correlated with eating behaviors. Some studies have shown that the personality types may affect eating behaviors which, in turn, affect choices of food intake [20, 21]. It is clear that inappropriate food choices and eating habits are related to obesity and weight gain [22] and controlling them may lead to proper weight management.

Maternal health, influences neonatal well-being. Maternal weight before and during pregnancy may influence the course of the pregnancy, fetal development, and the child’s health in both early and adult lives. A study that was conducted in Iran, showed inappropriate food intake among most of the pregnant women; however intake of different food groups had no relationship with weight gain during pregnancy [23]. The Institute of Medicine (IOM) has determined optimal range of weight gain during pregnancy based on women’s pre-pregnancy BMI [24]. Currently, less than one third of pregnant women have optimal weight gain during pregnancy and most of them gain excess weight [25, 26]. In this regard, a study in Iran confirmed pre-pregnancy BMI as a predictor of weight gain and found some socio-economic factors related to maternal weight gain, but psychological factors were not investigated [23]. A better understanding of factors related to inappropriate weight gain during pregnancy is necessary. The relationship between personality traits and weight gain, diet and eating behaviors of pregnant women has remained unknown. Finding the relationship between these items helps nutritional consultants to improve maternal education, especially in developing countries. To the best of our knowledge, no studies have investigated the effect of personality traits on dietary intakes and weight gain during pregnancy [27]. Therefore, this study was conducted in Iran to evaluate the association of mother’s personality trait with eating behaviors, food intake, maternal weight gain, and neonatal birth weight.

## Methods

### Study design

This study was a longitudinal and cross-sectional survey. All pregnant women were recruited at the second half of pregnancy period and were followed until delivery.

### Participants and recruitment

This study was conducted in Ahvaz, a city in South of Iran. Subjects were recruited from healthy pregnant women referred to Kurosh and Saa’di health centers, respectively representative of health centers in east and west of Ahvaz. The inclusion criteria were 18–45 years of age at the expected date of delivery, and subjects being at the 20th- 22nd gestational weeks. Since many pregnant woman experience morning sickness during the first months of pregnancy which may continue up to 3rd or 4th months, we decided to use the second half of pregnancy for the study protocol with the other reason being that most of the maternal weight gain occurs in the second half of pregnancy. Exclusion criteria were as follows: those having chronic infectious diseases, metabolic disorders, gestational diabetes, gestational hypertension, pre-eclampsia, eclampsia, twin pregnancy, preterm delivery, eating disorders (anorexia, bulimia), and emotional disorders (depression, stress and anxiety). In the first half of the pregnancy (the first 20 weeks), psychiatric evaluation is performed routinely using the Kessler Psychological Distress Scale (K10), for determination of depression and anxiety.

The sample size was calculated based on the relationship between personality traits of adults and BMI [28]. The following formula was used to calculate sample size. The correlation coefficient (r), α, and β were 0.17, 0.05 and 0.1 respectively. $n={\left[\frac{z_{1-\frac{\alpha }{2}}+{z}_{1-\beta }}{C}\right]}^2$, *C* = 0.5[ln(1 + *r*)/(1 − r)].

Considering the inclusion criteria and the calculated sample size, 360 pregnant women participated in the study.

### Instruments

Three valid and reliable questionnaires, (NEO Five-Factor Inventory, Dutch eating behavior questionnaire and a semi-quantitative food frequency questionnaire) were used to evaluate personality traits, eating behavior and food intake of participants respectively. All questionnaires were completed by trained interviewers at the first visit (after recruitment to the study). The interviewers explained about the questionnaires to the participants, before asking them to complete the questionnaires, also checked and resolved any vague recalls.

#### Personality traits questionnaire

NEO Five-Factor Inventory (NEO-FFI) by McCrae and Costa [15] was used to measure FFM personality domains. NEO-FFI is one of the most widely-used instruments to describe individual differences in five domains of personality traits (Neuroticism, Extraversion, Openness to Experience, Agreeableness, and Conscientiousness). Neuroticism is the tendency to experience negative emotions (anxiety, anger, and depression). Extravert people are active, sociable, and they search for stimulation. People with the Openness trait are unconventional, imaginative, creative, and artistically sensitive. Agreeableness is the tendency to be altruistic, trusting, modest, and cooperative. Conscientiousness individuals are strong-willed, reliable, persistent, and complying with rules and ethical principles [29].

#### Eating behavior questionnaire

Eating behaviors (restrained, emotional and external) were assessed using the Dutch eating behavior questionnaire (DEBQ) which has 33-items [30]. Ten items are included in the restraint scale which measures intentional restriction of eating in order to reduce or maintain weight. The DEBQ includes 13 items to evaluate the emotional scale and assesses eating due to emotional disorders such as anger, fear or anxiety, and not the physiological hunger. The DEBQ external scale (10 items) measures eating in response to external food-related stimuli, regardless of the internal state of hunger or satiety.

#### Food intake questionnaire

The whole diet was assessed using a validated and reliable semi-quantitative food frequency questionnaire (FFQ) that has been designed according to Iranian food guide pyramid [31]. The questionnaire included common foods typically consumed by Iranians. The standard serving sizes were defined in the questionnaire. Participants reported their average frequency of food intake over the past year. The frequency of intake was marked as times per day (e.g. bread), week (e.g. cheese), or month (e.g. fish). In addition, participants reported the serving size. The food frequency recall bias was limited by showing the participants the photographs of various portions and household or standard units. After completing the FFQ, the serving size was converted to grams and the reported amount of each food was converted to a daily intake value [32]. This questionnaire consisted of 45 food items. Some common foods in this questionnaire are as follows: milk, yoghurt, doogh (traditional yoghurt drink), ice cream and cheese consumption were considered in milk and dairy products group; red meat, poultry, fish, egg and legumes as proteins group; bread, rice and pasta as grains group; jam, confection, sugar, and soft drinks as sweets group; and fast foods, butter and oils as fats group.

#### Anthropometric assessment

Anthropometric indices, including weight gain during the second half of pregnancy as well as the infant’s weight, were measured by trained research staff and recorded in individual health cases. These data were collected from the health cases in health centers. The required amount of weight gain during pregnancy was determined according to the BMI before pregnancy (the weight before pregnancy was collected from the health case of mothers by self-report. The pre-pregnancy weight had been written in the first weeks of pregnancy, so mothers could recall their weight before pregnancy). The final subjects’ weight were taken at 38th week of gestation. Gestational weight gain was considered adequate in the second and third trimesters if the woman was within the range recommended by IOM based on pre-pregnancy BMI. According to IOM recommendation, the rate of weight gain during second and third trimesters for low and normal pre-pregnancy BMI was 0.5 kg and 0.4 kg per week, respectively. If pre-pregnancy BMI was between > 26.0–29.0 kg/m^2^, the recommended weight gain was 0.3 kg/week. For pre-pregnancy BMI more than 29, the rate of weight gain during second and third trimesters were not specified but the recommendation was that total weight gain must be less than 7.5 kg [24].

### Data analysis

Statistical analysis was performed with SPSS version 17.0 software. Data were expressed as means ±standard deviation (SD) for continuous variables or number (percentage) for categorical variables. The normality of data was analyzed using kolmongrov-smirnov test. Pearson correlation test was used to analyze the relationships between the personality traits, and eating behavior, food intake and anthropometric measurements. The One–way analysis of variances (ANOVA) was used to compare the mean of eating behaviors’ score, gestational weight gain, and birth weight of infants among different tertiles of personality traits’ score. Tukey test was used to compare which means differ from the rest. If distribution was not normal, related non-parametric test was used. Analysis of covariance was used to control the potential effect of some covariates such as length of gestation, pre-pregnancy BMI and maternal age. Predicting gestational weight gain and birth weight of infants by personality traits, eating behaviors and food intakes were analyzed using multinomial logistic regression. Reference group was considered adequate for both gestational weight gain and infant birth weight. For gestational weight gain, subjects’ weight gain were considered adequate if they were in line with IOM recommendation (mentioned in the previous section). Also birth weight between 2500 and 4000 g was considered adequate.

## Results

**Fig. 1:**
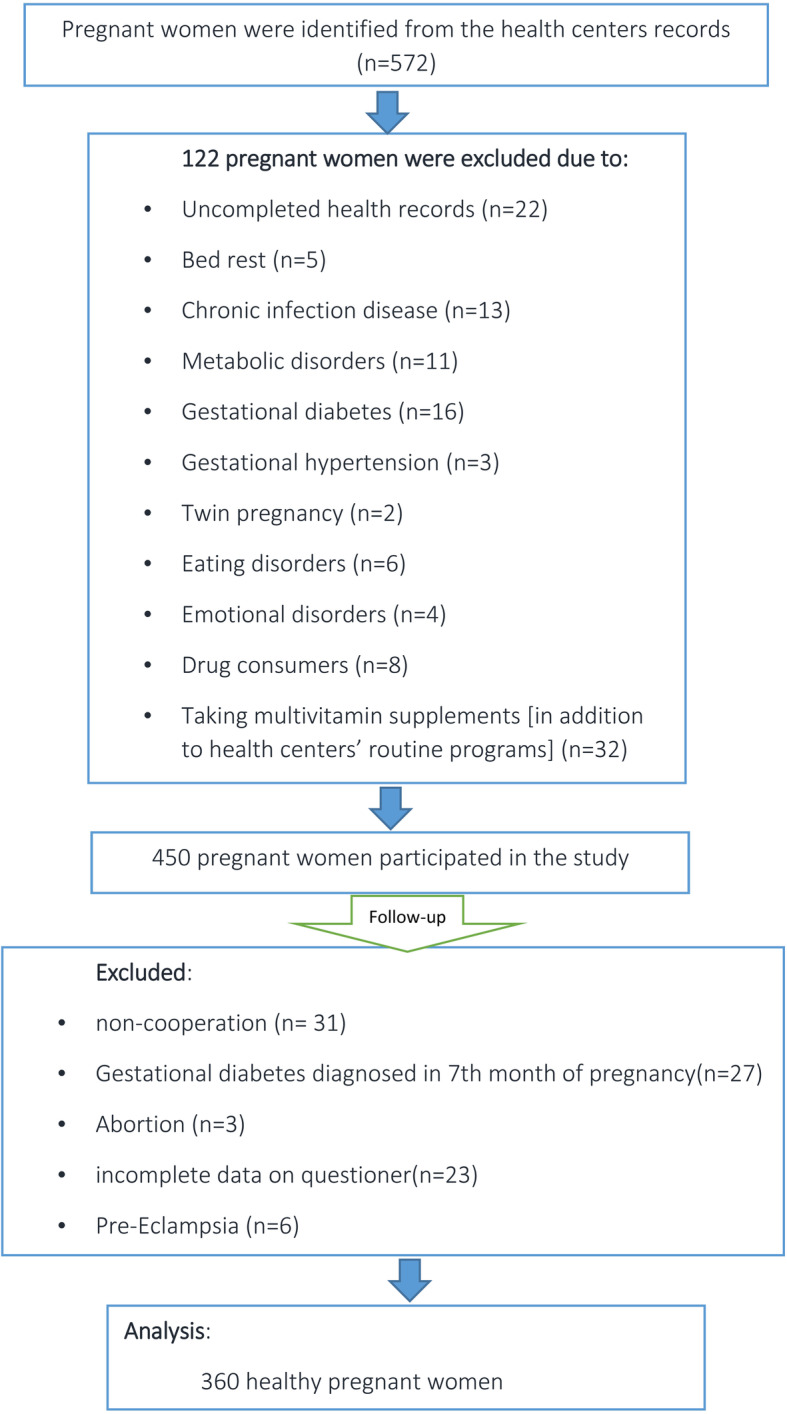
The flowchart of recruitment and follow-up of pregnant women

Figure 1 shows the subjects’ enrollment and follow-up for the study. In total, 360 women were included in the analysis. The characteristics of these women are described in (Table 1).

**Table 1:** Descriptive results for study population, demographics, personality traits, eating behaviors, dietary intake, weight gain and neonatal birth weight (*n* = 360)

Variables	Mean ± SD
Age (year)	28.46 ± 5.14
Pre-pregnancy BMI (kg/m^2^)	25.33 ± 3.7
Personality traits (score)	
Neuroticism	33.6 ± 6.16
Extraversion	40.5 ± 4.35
Openness	36.16 ± 3.37
Agreeableness	41.5 ± 3.48
Conscientiousness	41.15 ± 2.84
Eating behavior (score)	
Restrained eating	2.40 ± 0.5
Emotional eating	2.44 ± 0.5
External eating	2.33 ± 0.52
Dietary food (serving/day)	
Grains	12 ± 3.6
Dairy products	5.13 ± 1.95
Proteins	4.04 ± 1.68
Fruits	3.45 ± 1.09
Vegetables	2.09 ± 0.95
Total weight gain (kg)	12.7 ± 3.2
Second half of pregnancy weight gain (kg)	11.28 ± 3.27
Birth weight (kg)	3.1 ± 0.47

### Bivariate correlation

Pregnancy weight gain had positive correlation with birth weight of infants (*p* < 0.001). Pearson correlations suggest that personality factors are significantly correlated with eating behaviors and women’s dietary food intakes. Only the correlations between personality traits and eating behaviors and food groups were mentioned in (Table 2) to avoid its expansion.

**Table 2:** The relationship between major variables of the study, (*n* = 360)^a^

	Openness	Extraversion	Neuroticism	Conscientiousness	Agreeableness
Total weight gain	−0.02	− 0.04	− 0.02	−0.08	− 0.06
Birth weight	−0.05	− 0.07	−0.1	− 0.2^**^	−0.04
Grains	0.05	0.07	−0.08	0.11^*^	0.03
Dairy products	0.11^*^	0.02	0.12^*^	0.06	−0.15^**^
Proteins	−0.01	0.09	−0.04	0.09	0.07
Fruits	−0.04	−0.02	− 0.09	0.12^*^	0.06
Vegetables	0.11^*^	0.22^**^	−0.25^**^	0.16^**^	0.13^*^
Sweets	0.03	0.06	0.10^*^	0.04	0.01
Fats	0.04	0.21^**^	−0.11^*^	0.2^**^	0.03
Emotional eating	0.2^**^	0.12*	0.07	0.18^**^	−0.02
Restrained eating	0.12^*^	0.12^*^	0.05	0.11^*^	−0.05
External. eating	0.2^**^	0.11*	0.10^*^	0.17^**^	−0.06

^a^Presented as Pearson correlation coefficient (r); ^*^p < 0.05; ^**^p < 0.01

Neuroticism personality trait had a significant positive correlation with the consumption of ice cream (*p* = 0.001) and sweets but a significant negative correlation with the consumption of vegetables.

Extraversion had a significant positive correlation with restrained, external and emotional eating behavior as well as frequency intake of vegetables, fish (*p* = 0.023), legumes and eggs (*p* = 0.001), but had a significant negative correlation with fast food (*p* < 0.001) and red meat (*p* = 0.001) consumption. Openness to experience showed significant positive correlation with restrained, external and emotional eating behavior and frequency intake of vegetables, dairy products, and legumes (*p* = 0.001). However, it negatively correlated with the consumption of red meat (*p* = 0.024). Agreeableness had significant positive correlation with the frequency intake of vegetables and fish (*p* = 0.023) but was found to correlate negatively with the frequency intake of dairy products and ice cream (*p* = 0.01). Conscientiousness had a significant positive correlation with restrained, external and emotional eating behavior and the frequency intake of fruits, vegetables, legumes, and eggs (*p* = 0.001) but a negative correlation with frequency intake of red meat (*p* = 0.001) and confections (*p* = 0.002).

### Comparison of eating behaviors between levels of personality traits

The analysis of variance was used to examine the associations between personality traits and eating behavior, weight gain in pregnancy both in the second half and throughout the whole pregnancy period, and birth weight of infants. Emotional eating behavior of women with high neuroticism was significantly higher than those with moderate neuroticism (*p* = 0.02); and women with high neuroticism compared to moderate and low neuroticism gained significantly higher scores of external eating behavior (*p* < 0.001).

Women with moderate extraversion had significantly lower score of restrained eating compared to low and high scores of extraversion (*p* < 0.001); subjects with higher extraversion had higher scores of emotional eating behavior (*p* < 0.001).

Individuals with high openness to experience had significantly higher restrained, emotional and external eating scores compared to those with low or moderate openness (*p* < 0.001).

Women who scored high in conscientiousness were significantly higher in restrained eating behavior compared to the moderate group (*p* < 0.001). However, high conscientiousness women had higher scores of emotional and external eating behavior compared to women in low and moderate groups, respectively (*p* = 0.001). The results were shown in (Table 3).

**Table 3:** The mean and standard deviation of eating behavior scores, total and second half of pregnancy weight gain and birth weight of infants among different tertile groups of personality traits (*n* = 360)^a^

		Restrained eating (score)	Emotional eating (score)	External eating (score)	Total weight gain (kg)	Second-half weight gain (kg)	Birth weight (kg)
Personality traits	N	Mean(SD)					
**Neuroticism**							
Low	115	2.38(0.42)	2.44(0.47)ab	2.26(0.50)a	12.33(3.35)a	11.03(3.38)ab	3.12(0.48)ab
Moderate	149	2.36(0.53)	2.38(0.51)a	2.27(0.50)a	13.5(3.12)b	11.94(3.09)a	3.16(0.47)a
high	96	2.49(0.54)	2.56(0.50)b	2.48(0.53)b	12.1(3.26)a	10.57(3.24)b	2.99(0.43)b
*p*-value^#^		0.14	0.027^**^	0.002^**^	0.001^**^	0.004^**^	0.02^*^
**Extraversion**							
Low	100	2.45(0.54)a	2.43(0.47)ab	2.33(0.54)	12.89(3.12)	11.49(2.92)	3.09(0.46)
Moderate	142	2.29(0.45)b	2.36(0.49)a	2.27(0.44)	12.87(3.57)	11.2(3.68)	3.14(0.47)
high	118	2.50(0.51)a	2.56(0.51)b	2.39(0.58)	12.51(3.07)	11.21(3.04)	3.06(0.51)
*p*-value^#^		0.002^**^	0.004^**^	0.18	0.6	0.7	0.11
**Openness**							
Low	113	2.36(0.47)a	2.28(0.42)a	2.20(0.44)ab	12.69(2.91)	11.27(2.82)	3.09(0.4)
Moderate	159	2.36(0.47)ab	2.50(0.52)b	2.32(0.48)a	13.03(3.46)	11.36(3.45)	3.15(0.43)
high	88	2.55(0.57)b	2.56(0.53)b	3.50(0.63)b	12.35(3.41)	11.17(3.5)	3.02(0.51)
*p*-value^#^		0.007^**^	0.001^**^	0.001^**^	0.2	0.9	0.18
**Agreeableness**							
Low	91	2.39(0.64)	2.41(0.47)	2.32(0.56)	12.84(2.87)	11.23(2.73)	3.15(0.43)
Moderate	183	2.42(0.46)	2.45(0.49)	2.35(0.47)	12.9(3.41)	11.49(3.49)	3.08(0.46)
high	86	2.38(0.43)	2.46(0.54)	2.29(0.57)	12.36(3.43)	10.9(3.31)	3.11(0.51)
*p*-value^#^		0.74	0.75	0.71	0.4	0.38	0.49
**Conscientiousness**							
Low	90	2.38(0.52)ab	2.44(0.46)a	2.27(0.49)ab	12.79(3.18)ab	11.28(2.98)	3.25(0.43)a
Moderate	170	2.34(0.47)a	2.31(0.48)ab	2.24(0.51)a	13.13(3.29)a	11.52(3.29)	3.18(0.46)a
high	100	2.54(0.51)b	2.68(0.47)b	2.52(0.50)b	12.09(3.29)b	10.88(3.47)	2.96(0.48)b
*p*-value^#^		0.005^**^	0.001^**^	0.001^**^	0.04^*^	0.3	0.001^**^

^a^Data are presented as Mean (SD), ^#^ One way ANOVA, *p < 0.05, ^**^p < 0.01, Tukey post hoc analysis was done and different letters indicated the significant difference

### Comparison of birth weight in relation to levels of personality traits

Neonatal birth weight was lower in women with high neuroticism in contrast to those with moderate neuroticism (*p* = 0.017). Also, a significant difference was found in birth weight between women with low and high conscientiousness (*p* < 0.001), as well as moderate and high conscientiousness (*p* = 0.034), which means neonatal birth weight among women with high conscientiousness was lower than women with low or moderate conscientiousness (Table 3).

### Associations between personality traits, eating behaviors, food intake, and gestational weight gain

The multinomial logistic regression analysis was used to determine if personality traits, eating behaviors, or frequency intake of foods could predict gestational weight gain and neonatal birth weight.

The reference group for total weight gain, the second half of pregnancy weight gain, and neonatal birth weight was considered ‘adequate’. Table 4 shows the outputs of ‘insufficient’ compared to ‘adequate’. The outputs of ‘excessive’ compared to ‘adequate’ were not shown in the table due to the very low relation between them.

**Table 4:** Multinomial logistic regression for analysis of the associations of personality traits, eating behaviors and food intake with weight gain during pregnancy and neonatal birth weight (*n* = 360)

	Total weight gain				Second half weight gain				Birth weight			
	Insufficient^a^				Insufficient^a^				Low^a^			
	B	*p*	OR	CI	B	*p*	OR	CI	B	*p*	OR	CI
**Personality**												
Neuroticism	−0.023	0.212	1.04	0.98–1.09	−0.023	0.631	0.98	0.89–1.07	0.045	0.115	1.05	0.99–1.10
Extraversion	0.049	0.238	1.05	0.97–1.14	**0.162**	**0.015**	**1.18**	**1.03–1.34**	0.023	0.582	1.02	0.94–1.11
Openness	0.059	0.220	1.06	0.97–1.12	−0.169	0.051	0.84	0.71–1.01	0.043	0.372	1.04	0.95–1.15
Agreeableness	0.010	0.835	1.01	0.918–1.12	−0.034	0.680	0.97	0.82–1.14	0.058	0.237	1.06	0.96–1.16
Conscientiousness	0.073	0.197	1.08	0.96–1.20	−0.053	0.586	0.95	0.78–1.15	**0.183**	**0.001**	**1.20**	**1.07–1.34**
Eating behavior												
Restrained eating	−0.056	0.920	0.95	0.32–2.82	−0.891	0.338	0.41	0.07–2.54	−0.632	0.241	0.53	0.18–1.52
Emotional eating	**1.098**	**0.034**	**2.99**	**1.08–8.28**	0.80	0.372	2.22	0.38–12.8	**1.366**	**0.008**	**3.92**	**1.43–10.74**
External eating	−0.948	0.103	0.39	0.12–1.21	−0.706	0.50	0.59	0.6–3.84	−0.145	0.802	0.86	0.28–2.68
**Food intake**												
Grains	0.08	0.062	1.08	0.99–1.18	0.034	0.653	1.03	0.89–1.20	0.049	0.244	1.05	0.97–1.14
Dairy products	−0.033	0.696	0.97	0.82–1.14	−0.159	0.331	0.85	0.62–1.17	−0.023	0.780	0.98	0.83–1.15
Proteins	0.011	0.862	1.01	0.89–1.15	0.204	0.089	1.23	0.97–1.55	0.045	0.480	1.05	0.92–1.19
Vegetables	**−0.399**	**0.023**	**0.67**	**0.47–0.95**	**−0.83**	**0.006**	**0.41**	**0.22–0.77**	−0.325	0.057	0.72	0.51–1.00
Fruits	−0.067	0.676	0.93	0.68–1.28	−0.154	0.595	0.86	0.49–1.51	0.084	0.59	1.09	0.80–1.47
Fats	−0.023	0.938	0.97	0.55–1.73	**1.384**	**0.018**	**3.99**	**1.27–12.5**	−0.041	0.886	0.96	0.55–1.69
Sweets	−0.013	0.949	0.99	0.66–1.47	−0.072	0.837	0.93	0.47–1.85	0.215	0.260	1.24	0.85–1.80

^a^The reference category is adequate; *B* beta; *p p*-value; *OR* odds ratio; *CI* confidence interval. The effects of maternal age, pre-pregnancy body mass index and length of gestation were adjusted

The results of this table showed that women with extraversion were more likely to have insufficient weight gain during the second half of pregnancy.

The emotional eating behavior was associated with insufficient weight gain as well as low birth weight infants.

Subjects with increased frequency intake of fats and decreased vegetables were more likely to have insufficient weight gain.

### Associations between personality traits, eating behaviors, food intake, and birth weight

The risk of low birth weight infants was increased in pregnant women who had high scores in conscientiousness levels. This means that higher scores of conscientiousness was related to lower neonatal birth weight and that lower intake of vegetables could increase the chance of lower birth weight (Table 4).

## Discussion

The present study was conducted to examine the relationship between personality traits, eating behavior, dietary food intake, gestational weight gain and neonatal birth weight.

### Personality trait, eating behavior, food intake and gestational weight gain

In the current study, we tried to find associations between personality traits, eating behaviors, and dietary intakes with oscillations in weight during pregnancy in a longitudinal relation. Most pervious researches had focused exclusively on the relation between personality and BMI (primarily self-reported weights) [7, 21, 33]. Although weight gain during pregnancy occurs gradually, some individual differences may affect the rate and magnitude of weight gain. The results of our study revealed associations of extraversion, neuroticism and conscientiousness with weight gain during pregnancy. Extraversion predicted insufficient weight gain, which was inconsistent with data from non-pregnant extraversion women, who had a positive association with overweightness and an inverse association with underweight [34]. Another study on women 30–54 years of age from general population has reported higher scores of extraversion to be associated with obesity [35]. Extraverts are impulsive and expressive and we found them to be more emotional eaters. Also we found that emotional eating predicted more insufficient weight gain. A study conducted on obese people showed a relationship between emotional eating with lower extraversion [36]. Although this study was conducted in a different population, it seems that hormonal and psychological changes during pregnancy have effect on the extraversion ones since it could be implied that emotional eating during pregnancy was accompanied with non-proper food choices which led to insufficient weight gain, among extravert-emotional eater pregnant subjects.

The present study showed the positive relationship of conscientiousness with restrained eating behavior, in contrast with other studies [28, 36]. In addition, subjects in the third tertile of conscientiousness were gained the lower weight. Our results were in line with previous study that revealed people with low score of conscientiousness, gained more weight [37]. It seems the positive relationship between restrained eating behavior and conscientiousness is associated with the lower weight gain among the most conscientious subjects of our study.

In this study, women with high scores of neuroticism had a tendency to gain less weight, in line with another study in which neuroticism had an inverse association with overweightness [34]. Another study, however showed no association between neuroticism and obesity [35]. The results of our study confirmed a negative correlation between frequency intake of vegetables and the score of neuroticism, and less frequent intake of vegetables (as a representative of healthy diet) predicted insufficient weight gain. Another finding was that high scores of neuroticism were related to high scores of emotional and external eating, and in this study, emotional eating predicted insufficient weight gain. Then it could be explained why neurotic women experienced lower weight gain during pregnancy. The finding of this study, showed unexpected results about some types of personality, eating behaviors and weight gain during pregnancy, when they compare with other studies which were conducted on general population or obese people [36]. Although some other studies found no relationship between any eating behavior and gestational weight gain [33]. In the meantime, other studies, showed less restrained eating behavior during pregnancy but it had not any relationship with weight gain [38]. So it would be interesting to compare the relationship between eating behaviors, different food intakes and weight gain, at pre-pregnancy state and during pregnancy, in future studies.

Here we would have a look on our findings about food intake and weight gain. We showed that a more frequent intake of fat could predict insufficient weight gain; inconsistent with findings in other studies which showed that fried foods were directly associated with excessive weight gain [39]. In contrast, our findings revealed that decreased frequency intake of vegetables might predict insufficient weight gain. Other studies did not observe any risk of excessive gestational weight gain in relation to consumption of vegetables [39–41]. It could be explained that a more frequent intake of vegetables and a less frequent intake of fats or sweets, are more appropriate and would result in adequate weight gain.

### Personality trait, eating behavior and neonatal birth weight

To the best of our knowledge, to date, two studies on general population and couples have assessed all the FFM domains and changes in BMI over a period of time [7, 25] and our study is the first to explore the association between the personality traits with gestational weight gain. It is also the first one dealing with the way FFM contributes to key criteria of birth weight. The results indicated lower birth weight of infants among mothers with high score of neuroticism and conscientiousness personality traits. Apparently, this finding is related to maternal weight gain because mothers with high score of neuroticism or conscientiousness experienced less weight gain.

### Strengths and limitation of the study

The limitation of the study was the employment of multiple questionnaires to be filled by the participants, which might have been boring them. Therefore, it was difficult to use a long food frequency questionnaire to determine dietary pattern. Also, different delivery times among the subjects forced the researchers to record birth weights only from infants’ health cards rather than weighting the infants themselves. Finding the association of personality traits with maternal weight gain and birth weight of infants, was the major point of the study. However, it would be suggested to evaluate gestational growth in the second and third trimester and hematochemical parameters, as additional variables in future studies. Another important point was the use of a dietary food intake questionnaire in accordance with Iranian food guide pyramid to evaluate the consumption of major components of food groups and subgroups. Most of the previous studies, however, only reported the association of energy intake during pregnancy with gestational weight gain. Also, this research was done as a longitudinal study in which the subjects were followed from the second half of their pregnancy until delivery.

## Conclusion

The findings of the study revealed the relationship of personality trait with weight gain during pregnancy. It seems identification of the individual personality traits can have positive effect on health and nutritional education especially during the important periods of life such as pregnancy and lactation. Thus this could be studied as a new approach in nutritional consultation during pregnancy, in future. In this regard, studying the eating behaviors and food pattern before pregnancy and their changes during pregnancy, may be helpful in identifying the exact relationship between eating behaviors and personality traits at pregnancy.

## Data Availability

Data of the present research is available by contacting the corresponding author on reasonable request.

## Abbreviations

ANOVA: One–way Analysis of Variances
BMI: Body Mass Index
DEBQ: Dutch Eating Behavior Questionnaire
FFM: Five-Factor Model
FFQ: Food Frequency Questionnaire
IOM: Institute of Medicine
NEO-FFI: NEO Five-Factor Inventory
SD: Standard Deviation
